# Body image and weight perceptions in relation to actual measurements by means of a new index and level of physical activity in Italian university students

**DOI:** 10.1186/1479-5876-12-42

**Published:** 2014-02-11

**Authors:** Luciana Zaccagni, Sabrina Masotti, Roberta Donati, Gianni Mazzoni, Emanuela Gualdi-Russo

**Affiliations:** 1 Department of Biomedical and Specialty Surgical Sciences, University of Ferrara, Corso Ercole I d’Este 32, 44121 Ferrara, Italy; 2 Center of Biomedical Studies Applied to Sport, University of Ferrara, Via Gramicia 35, 44123 Ferrara, Italy

**Keywords:** Body image, Physical activity, Weight status, FID, FAI

## Abstract

**Background:**

Body image perception depends on anthropometric and psychological factors. Body dissatisfaction is influenced by the socio-cultural environment and is associated with eating disorders and low self-esteem. This study examined the body image perception, the degree of dissatisfaction and the weight status perception inconsistency in relation to sex, weight status and amount of physical activity in a sample of university students.

**Methods:**

The participants were 734 university students (354 females aged 21.5 ± 2.9 yrs and 380 males aged 22.1 ± 3.6 yrs) recruited from the second year of the Sport Sciences degree program. A self-administered questionnaire was used to acquire socio-demographic and sport participation information. Height, weight, BMI and weight status were considered for each subject. Body image perception was assessed by a silhouette matching technique. A new index, FAI (Feel status minus Actual status Inconsistency), was used to assess weight status perception inconsistency.

**Results:**

A large proportion of the sample had normal weight status. On average, females chose as feel status a significantly higher figure than the males (4.7 versus 3.8) and they would have liked to have a significantly thinner figure than the males (3.4 versus 3.6). Therefore, the mean FID (Feel minus Ideal Discrepancy) values (positive in both sexes) were significantly higher in females than in males, meaning higher dissatisfaction. The mean FAI values were positive in females and negative in males, indicating a tendency of the women to overestimate their weight status and of the men to underestimate it. Men were more physically active than women. Less active women showed significantly lower body weight and BMI than more active women. Men less engaged in physical activity showed significantly higher FID than more active men.

**Conclusions:**

These results show greater dissatisfaction and higher weight status perception consistency in females than in males among Italian university students examined. Our findings suggest that the FAI index can be very useful to evaluate the perceived weight status by body image in comparison to actual weight status assessed anthropometrically.

## Background

Body image was defined as “a person’s perceptions, thoughts, and feelings about his or her body” by Grogan [1] and it depends on various factors: psychological components and socio-cultural influences such as family, peers, and ethnicity [2-6]. No less important are the mass media which generate aesthetic ideals influencing the perception of one’s image and leading to a tortuous search for the ideal body: this creates real pressure that leads to internalization of a beauty ideal and to an inevitable desire to conform to it [7].

As body dissatisfaction is a risk factor for eating disorders [8], the importance of assessing and reducing this dissatisfaction must be emphasized. Inclusion of strategies that reduce body dissatisfaction and increase body esteem could improve treatment effects [9]. In particular, regular participation in physical activity confers many positive health outcomes in young people, such as reduced risk of coronary heart disease, hypertension, depression and obesity [10,11].

In addition to the physical benefits, physical exercise also has psychological benefits, particularly in women. However, while fitness and health motivations may be associated with positive consequences of physical exercise for individuals with low body dissatisfaction, greater endorsement of both fitness and health motivations, as well as appearance and weight motivations, are associated with even greater state body dissatisfaction in women categorized as high body dissatisfied [12].

In general body, weight and shape satisfaction decreases with increasing BMI and the search for desired physical form involves a wide range of behaviors and activities such as dieting and physical exercise [13,14]. Dieting is more frequent among women than among men [15], who tend to practice physical exercise rather than diet to change the look of their bodies and to lose weight [16,17]. Moreover, improving body image, particularly by reducing its importance in one’s personal life, plays a role in enhancing eating self-regulation during weight control in women [18].

Although research generally demonstrated that high motivators for physical exercise participation are weight management, appearance and body dissatisfaction [14,19], the relationship between level of satisfaction and physical activity is still unclear: men practice it more than women according Neumark-Sztainer et al. [20], probably because men are more satisfied with their body. A negative body image may act as a barrier to physical participation and may be involved in social physique anxiety linked to real or unreal negative physical evaluation [13,21].

Nevertheless other studies have shown that women, whether active or inactive, are at greater risk of body dissatisfaction and disordered eating than men [22-25]: those who perceive themselves as overweight are more likely to physical exercise to lose weight than those who do not feel themselves overweight, while weight misperception among overweight and obese adults is associated with less likelihood of interest in or attempts at weight loss and less physical activity [26].

Although many studies have been carried out in this field, it is important to proceed further in this direction to better understand the many facets of the concept of body image and its perception. In particular there is a need to study lifestyle factors and their association with body image and perception in young adults.

The aim of the present study was to examine the body image perception, the degree of dissatisfaction and the weight status perception inconsistency in relation to sex, weight status and amount of physical activity in a sample of university students. A major goal was to define and validate a new index to assess weight status perception in comparison to actual weight status. The research analyzed (i) to what extent students are concerned about their general body image, and the eventual discrepancy with ideal body image; (ii) the relationship between body image perception, gender, weight status and practice of physical activity; (iii) the consistency between weight status perception and actual measurements. The last point must be emphasized as it represents an important topic that has been poorly addressed.

## Methods

### Sample and procedures

A cross-sectional survey was conducted on a volunteer sample of 734 Italian university pre-selected students (354 females aged 21.5 ± 2.9 yrs and 380 males aged 22.1 ± 3.6 yrs) from the Sport Sciences degree program (Faculty of Medicine, University of Ferrara, Italy).

The students were measured in the Anthropometric Laboratory of the University of Ferrara during the tutorials for the Anthropometry and Ergonomics course in the second year of the Sport Sciences degree program (survey years: 2002-2011). After explaining the purpose of the research project, 91% of students of both sexes agreed to participate in the study. The research protocol was approved by the Ethic Committee for Biomedical Research of the University of Ferrara, and all participants provided written informed consent.

A self-administered questionnaire was used to acquire socio-demographic information (sex, age) and sport participation information (hours/week, sport activities).

### Variables

#### Anthropometrical measures

Height and weight were measured by trained examiners. Body mass was measured to the nearest 0.1 kg and standing height to the nearest 0.1 cm using a balance scale and a wall-mounted stadiometer (Magnimeter, Raven Equipment Limited, UK), respectively, with the subject barefoot and clothed appropriately, according to standardized procedures [27]. Body mass index (BMI) was calculated as body weight/height^2^ (kg/m^2^) and was used to assess each student’s weight status. The World Health Organization (WHO) cutpoints [28] were used to classify subjects as underweight, normal weight, overweight and obese (actual status encoded respectively as nutritional status 1, 2, 3 and 4). As there were very few obese subjects (only one female and 15 males), they were included in the overweight group for further data analysis.

#### Body image assessment

Body image perception was assessed by a silhouette matching technique. Nine male and female figures with increasing body weight from figure 1 to figure 9 were shown to the students [29]. The subjects indicated which figure best represented how they currently looked (feel) and how they ideally wanted to look (ideal), which figure was their opposite sex ideal and which figure was the same sex ideal for the opposite sex.

Body dissatisfaction was calculated as Feel minus Ideal Discrepancy (FID) [30] with negative scores meaning the desire to be fatter and positive ones meaning the desire to be thinner. We devised the index FAI (Feel weight status minus Actual weight status Inconsistency), to assess the weight status perception inconsistency using the silhouette matching technique as a proxy to verify if there was or was not a realistic weight status perception in the subject on the basis of body size assessment (BMI) and the feel figure. The FAI was computed by subtracting the conventional code assigned to the actual weight status of the subject (code: 1 for underweight status, 2 for normal weight status, 3 for overweight status and 4 for obese status) from the one corresponding to her/his feel figure according to the following correspondence: figures 1 and 2 match feel status 1; figures 3, 4 and 5 match feel status 2; figures 6 and 7 match feel status 3; figures 8 and 9 match feel status 4.

The FAI scores range from -3 to +3: negative FAI values mean weight status underestimation, positive FAI values mean weight status overestimation and a FAI score of 0 means a realistic perception of one’s weight status.

#### Physical activity measures

The frequency of physical activity of each subject was determined on the basis of hours of training during a typical week, as declared by the subject. The effect of the practice of physical activity on body image perception was assessed in the sample divided into three tertiles: 1) subjects with poor physical activity (absent or low), 2) subjects with medium physical activity and 3) subjects with high physical activity. The 1^st^ tertile included females practicing physical activity ≤ 2 hours/week and males < 6 hours/week; the 3^rd^ tertile included females and males practicing physical activity ≥ 6 hours/week and ≥ 8 hours/week, respectively.

### Statistical analyses

All analyses were performed using “Statistica” for Windows, Version 11.0 (StatSoft Italia srl, Padua, Italy). Data for males and females were analyzed separately. The results were expressed as mean values and standard deviations (SD). Comparisons between two independent groups were performed using Student’s t-test for traits with normal distribution and the non-parametric Mann-Whitney U test for traits with non-normal distribution. Comparisons between two dependent variables were performed using paired-sample tests (Wilcoxon test) to assess differences in non-normally distributed data (between item responses). The Kruskal-Wallis one-way analysis of variance by ranks ANOVA was used to analyse main effects for body image perception in relation to BMI category. Pearson’s Chi-square test was used to compare categorical data.

The level of p < 0.05 was considered significant.

## Results

Table 1 shows the anthropometric characteristics and body image assessment of the sample by sex. On average, females were significantly shorter, lighter and had a lower BMI than males. A large proportion of the sample had normal weight status (80.9% in females and 71.7% in males), while the 13.5% of females and the 28.3% of males were overweight or obese. No male student was underweight, compared to 5.6% of females.

**Table 1:** Sample characteristics by sex

**Trait**	**Males**	**Females**	***P *** **value**
Height (cm)	177.6 ± 6.3	163.9 ± 6.0	<0.0001
Weight (kg)	75.6 ± 10.2	56.7 ± 8.2	<0.0001
BMI (kg/m^2^)	24.0 ± 2.8	21.8 ± 2.6	<0.0001
Feel figure	3.8 ± 1.6	4.7 ± 1.6	<0.0001
Ideal figure	3.6 ± 1.2	3.4 ± 1.2	0.0158
FID	0.17 ± 1.28	1.33 ± 1.27	<0.0001
FAI	-0.37 ± 0.55	0.21 ± 0.54	<0.0001
Opposite sex ideal	3.7 ± 1.1	3.7 ± 1.1	0.6752
Same sex ideal for the opposite sex	3.5 ± 1.1	3.3 ± 1.1	0.0010

Regarding the body silhouette chart, the females chose as feel a significantly higher figure than the males and they would have liked to have a significantly thinner figure than the males. Therefore the mean FID values (positive in both sexes) were significantly higher in females than in males, meaning higher dissatisfaction. Only 13% of the females were satisfied (FID = 0). Most of them (81%) would have liked to be thinner (FID > 0); indeed 42% and 23% of these females would have liked to be more slender by one or two figures, respectively. The remaining 6% would have liked to be more robust (FID < 0) than how they perceived themselves. Male students were more satisfied with their body image perception than females: 33.3% were satisfied (FID = 0), 28.3% would have liked to be more robust and 38.4% would have liked to be more slender than how they perceived themselves.

Moreover, regarding body dissatisfaction (FID) according to weight status of the subject, normal weight men were more satisfied with their body (38.3%) than underweight (30.4%) and overweight (16.1%) men (p < 0.0001), while 33.3% of underweight women were more content with their body image compared to 15% of normal weight and 3.5% of overweight women (p < 0.0001).

Both sexes showed a significant preference for thinner bodies: the mean value of ideal figure was significantly lower than the feel figure (p = 0.0198 for males and p < 0.0001 for females).

The mean FAI value was positive in females and negative in males indicating a tendency of women to overestimate their weight status and of men to underestimate it. Women had a more realistic body image perception than men (70% vs 58.4% respectively): 25.3% of women overestimated their weight status and only 4.7% of them underestimated it, while 2.6% of men overestimated their weight status and 39% of them underestimated it (p < 0.0001).

Men and women agreed on the selection of their opposite sex ideal; in fact, on average they chose the same figure. However the students thought that the same sex ideal for the opposite sex was significantly thinner than it actually was (in women’s opinion the same sex ideal for the opposite sex was 3.3 ± 1.1 vs 3.7 ± 1.1 of men’s actual opposite sex ideal p < 0.0001 ; in men’s opinion the same sex ideal for the opposite sex was 3.5 ± 1.1 vs 3.7 ± 1.1 of women’s actual opposite sex ideal p = 0.0141).

Tables 2 and 3 show the results of comparisons among BMI categories on the salient variables of body image perception in males and females, respectively. All the considered traits were significantly different, with the exception of the figure selected for the same sex ideal for the opposite sex by men.

**Table 2:** Body image assessment in male sample by weight status

**Trait**	**18.5 **≤ **BMI <25**	**BMI **≥ **25**	***P *** **value**
Feel figure	3.2 ± 1.2	5.3 ± 1.5	<0.001
Ideal figure	3.4 ± 1.1	4.2 ± 1.2	<0.001
FID	-0.21 ± 1.12	1.16 ± 1.14	<0.001
FAI	-0.27 ± 0.51	-0.63 ± 0.56	<0.001
Opposite sex ideal	3.6 ± 1.1	3.9 ± 1.1	0.049
Same sex ideal for the opposite sex	3.5 ± 1.0	3.6 ± 1.2	0.383

**Table 3:** Body image assessment in female sample by weight status

**Trait**	**BMI < 18.5**	**18.5 **≤ **BMI < 25**	**BMI **≥ **25**	***P *** **value**
Feel figure	2.6 ± 0.9	4.6 ± 1.4	6.6 ± 1.1	<0.001
Ideal figure	2.5 ± 0.7	3.3 ± 1.2	4.4 ± 1.1	<0.001
FID	0.08 ± 0.88	1.27 ± 1.24	2.22 ± 1.03	<0.001
FAI	0.44 ± 0.51	0.23 ± 0.53	0.02 ± 0.54	0.010
Opposite sex ideal	3.0 ± 1.2	3.7 ± 1.0	4.1 ± 1.2	0.001
Same sex ideal for the opposite sex	2.9 ± 0.6	3.2 ± 1.1	3.6 ± 1.0	0.020

In both sexes the mean figures increased with the increasing of BMI. The mean FID values also increased with increasing BMI in both men and women. In particular, normal weight males had a negative mean FID value, meaning that they would have liked to be more robust than they perceived themselves, while overweight males had a positive FID mean value meaning that they would have liked to be thinner than they perceived themselves. No group of women had negative mean FID value, but the value increased from the underweight condition to overweight/obesity.

Females showed positive mean FAI values, although they decreased with increasing weight status, indicating a general trend of this sex to weight status overestimation but also greater awareness of one’s weight status in overweight subjects. In contrast, there were negative mean FAI values in males which decreased with increasing weight status, indicating weight status underestimation that worsened with increasing BMI.

Table 4 shows the percentages of subjects of both sexes for choice of figure as feel, as ideal, as opposite sex ideal and as the same sex ideal for the opposite sex, as well as satisfaction (FID = 0) and weight status perception consistency (FAI = 0). Body images F5 and F3 were most frequently selected as feel in females and in males respectively. F3 and F4 were most frequently selected as ideal in females and in males respectively. F3 was most frequently selected as opposite sex ideal and as same sex ideal for the opposite sex in both sexes. Females and males who perceived themselves as F2 and F4, respectively, had the highest percentage of satisfaction (over 44%). Females and males who perceived themselves as F4 and F3, respectively, had the highest percentage of weight status perception consistency (95.7% for females and 88.7% for males).

**Table 4:** Percentages of males and females selecting each figure in body image assessment

	**Body image figures**								
	**F1**	**F2**	**F3**	**F4**	**F5**	**F6**	**F7**	**F8**	**F9**
**Feel**									
Males	4.9	16.4	27.5	21.6	12.9	9.4	5.4	1.6	0.3
Females	0.9	5.2	19.3	20.7	21.3	17.2	11.8	3.4	0.3
**Ideal**									
Males	1.1	16.9	29.2	31.9	14.4	4.9	1.4	0	0.3
Females	2.6	21.8	33.3	21.3	15.8	5.2	1.7	0	0
**FID = 0**									
Males	5.6	37.3	36.4	44.3	32.6	21.2	15.8	0	0
Females	0	44.4	22.4	9.9	13.9	6.8	0	0	0
**FAI = 0**									
Males	5.9	1.7	88.7	74.7	52.3	74.2	50.0	83.0	0
Females	33.3	52.9	90.8	95.7	92.9	21.4	48.6	9.1	0
**Opposite sex ideal**									
Males	0.8	7.9	42.3	20.9	22.5	4.6	0.8	0	0.3
Females	1.1	8.9	38.8	27.6	17.8	5.5	0.3	0	0
**Same sex ideal for the opposite sex**									
Males	1.6	15.7	34.3	29.7	15.1	3.0	0.5	0	0
Females	1.4	23.6	40.8	18.1	13.2	2.9	0	0	0

Men were more physically active than women, practicing a higher weekly amount of physical activity (6.7 ± 4.2 hrs/week vs 4.2 ± 3.8 hrs/week; p < 0.0001); only 28 males (7.4%) but almost one fourth of females (83 subjects, 23.4%) did not practice any sport activity. To verify the effect of physical activity on body satisfaction, we compared data of the 1^st^ tertile with those of 3^rd^ tertile in both sexes (Table 5). Less active women showed significantly lower body weight and BMI than more active women. Men who engaged in physical activity for fewer hours (1^st^ tertile) showed significantly higher FID and they believed that a thinner male was more attractive to females than men who engaged in physical activity for many hours (3^rd^ tertile).

**Table 5:** Body image assessment by sex and amount of physical activity

	**Females**			**Males**		
**Trait**	**I**^**st **^**Tertile**	**III**^**rd **^**Tertile**	***P *** **value**	**I**^**st **^**Tertile**	**III**^**rd **^**Tertile**	***P *** **value**
Height (cm)	163.5 ± 5.9	164.4 ± 6.6	0.2835	177.2 ± 6.2	178.1 ± 6.3	0.2731
Weight (kg)	57.9 ± 8.8	60.3 ± 8.0	0.0198	76.3 ± 11.3	74.9 ± 8.8	0.4221
BMI (kg/m^2^)	21.6 ± 2.9	22.3 ± 2.4	0.0169	24.3 ± 3.3	23.6 ± 2.2	0.1557
Feel figure	4.7 ± 1.6	5.0 ± 1.6	0.2546	3.8 ± 1.6	3.8 ± 1.6	0.9962
Ideal figure	3.5 ± 1.2	3.4 ± 1.3	0.7357	3.5 ± 1.3	3.8 ± 1.2	0.0916
FID	1.26 ± 1.32	1.55 ± 1.21	0.1214	0.33 ± 1.28	0.03 ± 1.27	0.0493
FAI	0.23 ± 0.53	0.25 ± 0.55	0.8892	-0.41 ± 0.58	-0.33 ± 0.53	0.3788
Opposite sex ideal	3.7 ± 1.1	3.7 ± 1.1	0.9794	3.7 ± 1.3	3.8 ± 1.0	0.5082
Same sex ideal for the opposite sex	3.3 ± 1.2	3.2 ± 1.0	0.6965	3.3 ± 1.1	3.7 ± 1.1	0.0245

## Discussion

In this study we examined the body image perception, degree of dissatisfaction and weight status perception inconsistency in relation to sex, weight status and amount of physical activity in a sample of Italian university students. According to the literature, the university population is regarded as a convenient sample for the study of health in young adults [31].

The comparison between ideal figure and feel figure showed that both men and women prefer ideals of thinness. The choice of a thin ideal body, characteristic in European countries and particularly in Italy, Greece, and in France [32], may lead to a body image disturbance and a consequent condition of dissatisfaction. Dissatisfaction with one’s body size is influenced by the socio-cultural environment, contributing to stresses of modern life, and it is associated with the current morbi-mortality [33]. Body image dissatisfaction affects both sexes and it is influenced by both culture and society, including mass media images often favoring models close to those of people suffering from anorexia [34,35].

Previous studies have shown that men and women differ in the perception of their body image and in dissatisfaction with their body [3,16]. In our study, body dissatisfaction (FID) increased with increasing BMI; the increase in FID was more accentuated in women (+2.14 from underweight to overweight women) than in men (+0.95 from underweight to overweight men).

Our findings confirm the typical female behavior derived from the cultural and social pressure to be thin with possible leading to a negative impact on quality of life, depression and low self-esteem [36,37]. In general, the female students of our sample tended to overestimate their weight status, while male students tended to underestimate it, in accordance with other studies [4,32,34,38].

We have proposed and used the FAI index, to measure weight status perception inconsistency. Females showed positive mean FAI values which decreased with increasing weight status (from +0.44 to +0.02), indicating a general trend to weight status overestimation but also greater awareness of weight status in overweight subjects. Instead the males showed negative mean FAI values which also decreased with increasing weight status (from -0.27 to -0.63), indicating weight status underestimation which worsened with increasing weight status. This index also highlights the health risks resulting from incorrect body image perception in underweight females and overweight/obese males: for example, underweight women who perceive themselves as overweight may develop negative behaviors such as unnecessary dieting and binge eating disorders.

In this study, females had a more consistent body image perception than males: in the overweight category, females were more likely than males to perceive themselves as overweight. This shows that men probably pay less attention to their nutritional status or they deny that it is a problem more than women; the consequence of this behavior may be poor preventive action against chronic degenerative diseases associated with obesity [33,39,40]. Most young adults are not vulnerable to these illnesses until they personally experience them [41]. Young adult males are more likely to be unaware of their body weight status than women [39,42] and, in particular, men are less critical of their body size than women, perceiving it as normal. Males often perceive themselves as smaller than they are, due to a desire for a more muscular body [4,34]. Instead, the women’s behavior could reflect environmental factors associated with the development of eating disorders. Women with low self-esteem are most inclined to beauty ideal internalization, which in turn increases the probability of restrictive behavior [43].

A sex difference also appeared in the percentage of individuals who were satisfied with their weight status (33.3% of males compared to 13% of females). This finding is consistent with literature reports suggesting that men are more satisfied with their current weight status than women [3,32]. Dissatisfaction with one’s body image among women was probably due to the female perception that males prefer a thin ideal figure of them. In our study, women were asked to choose the female figure they believed to be the opposite sex ideal for men, while men had to choose the male figure they felt would be the opposite sex ideal for women. Both the women and men felt that the opposite sex would desire a thinner ideal than it actually does, especially the females, in fact the men wanted a more curvy woman and the women a more muscular man. These findings suggest that men’s body image derives from a perceived lack of muscles, while women’s body image is due to perceived excess weight (o fat mass). It should be stressed that this ambiguity may depend on the fact that the figures furnish a uni-dimensional measure, unable to distinguish whether the increase of their dimensions is due to fat or to muscularity [44]. Moreover in some cases (e.g. athletes) the BMI could give an incorrect mistaken indication on nutritional status.

The sex difference in the practice of physical activity was particularly significant in this sample, with a triple rate of sedentary individuals in women compared to men. However the education towards an active lifestyle received during university studies probably helped to reduce the proportion of sedentary individuals in this sample, since it was much lower than the proportion recorded in 2006 in all Italian citizens over the age of 3 years (M: 36.2%, F: 45.2%) [45]. Furthermore, although the Italian survey indicated a negative trend of sport with age after 14 years, the weekly hours of activity in the examined university students are higher on average than those of a sample of 14-year-olds from the same region (4.7 ± 2.7 hours in males and 3.7 ± 2.5 hours in females) [46].

We also explored the relationship between body satisfaction and physical activity. Regular physical activity leads to physical and psychological benefits for individuals well-being. In theory, individuals who feel dissatisfaction are more likely to engage in behaviors to fight the discomfort. In our study, more active men were more satisfied than less active men. This is generally in agreement with other studies examining these associations in youth [3,21]; in contrast Douthitt [47] found positive associations between body satisfaction and physical activity in girls but not in boys. In our sample, even though the more active females were significantly heavier and had higher BMI than the less active ones, there were no differences in the degree of satisfaction; this was probably because the greater body mass of the former was fat free mass and not fat mass due to the greater amount of physical activity. For the same reasons, in the female sample we could not confirm literature data showing that body image perception is a strong predictor of adoption of healthy behavior, such as physical activity, which might prevent or control weight in young women [48]: no significant association between desired body image and physical activity was observed in these female students.

Some limitations must be considered when interpreting the findings of our study. First, data collection has continued over time and so there could be a shifting in bodily perception. Second, comparisons with other studies are difficult because they used different measures of body image (different silhouette charts, questionnaires) and physical activity. Finally, there were different types of physical activity in the sample, and thus the experiences of some participants might differ from those of students enrolled in fitness courses.

As it is difficult to determine if there is a relationship between body satisfaction and physical activity in women and to define the role of less physical activity in men (cause or effect of dissatisfaction), further studies are necessary to clarify the complex phenomenon of body image. Moreover, additional studies on body composition assessment could provide information about the relationship between fat mass and amount of physical activity.

## Conclusions

The results of this study confirmed the greater dissatisfaction and higher weight status perception consistency in females than in males in the Italian university students we examined, and described a new index for the assessment of body image. This research evidenced the dissatisfaction and the discrepancy with ideal body image in the examined sample (except the most active males) in particular in the female sex, in overweight individuals, with lower levels of physical activity.

Regarding the consistency between weight status perception and actual measurements, our findings suggested that the proposed FAI index could be adopted to accurately evaluate perceived weight status by body image in comparison to actual weight status assessed anthropometrically.

## Competing interests

The authors declare that they have no competing interests.

## Authors’ contributions

LZ, and EGR conceived the study. SM and LZ performed the statistical analysis. SM, and DR participated in data collection, organization, and drafted the manuscript. GM participated in drafting the manuscript and interpretations of the results. LZ and EGR helped in interpret the results and participated in drafting the final version of the manuscript. All authors read and approved the final manuscript.
